# A 3D Elastoplastic Constitutive Model Considering Progressive Damage Behavior for Thermoplastic Composites of T700/PEEK

**DOI:** 10.3390/ma17133317

**Published:** 2024-07-04

**Authors:** Weigang Fu, Huanjie Xiong, Zhe Liao, Junchi Ma, Yaoming Fu, Bin Wang

**Affiliations:** 1 College of Aviation Engineering, Civil Aviation Flight University of China, Guanghan 618330, China; jiaodafwg@126.com (W.F.); zhe_liao@126.com (Z.L.); junchi_ma2021@163.com (J.M.); ymfo@163.com (Y.F.); 2 Department of Mechanical and Aerospace Engineering, Brunel University London, Uxbridge UB8 3PH, UK

**Keywords:** CFRPs, T700/PEEK, two-parameter, three-dimensional elastoplastic damage, constitutive model, quasi-Newton method, Langmuir function, LaRC05 criterion

## Abstract

Due to their excellent mechanical properties, the carbon fiber-reinforced polymer composites (CFRPs) of thermoplastic resins are widely used, and an accurate constitutive model plays a pivotal role in structural design and service safety. A two-parameter three-dimensional (3D) plastic potential was obtained by considering both the deviatoric deformation and the dilatation deformation associated with hydrostatic stress. The Langmuir function was first adopted to model the plastic hardening behavior of composites. The two-parameter 3D plastic potential, connected to the Langmuir function of plastic hardening, was thus proposed to model the constitutive behavior of the CFRPs of thermoplastic resins. Also, T700/PEEK specimens with different off-axis angles were subjected to tensile loading to obtain the corresponding fracture surface angles of specimens and the load–displacement curves. The two unknown plastic parameters in the proposed 3D plastic potential were obtained by using the quasi-Newton algorithm programmed in MATLAB, and the unknown hardening parameters in the Langmuir function were determined by fitting the effective stress-plastic strain curve in different off-axis angles. Meanwhile, the user material subroutine VUMAT, following the proposed constitutive model, was developed in terms of the maximum stress criterion for fiber failure and the LaRC05 criterion for matrix failure to simulate the 3D elastoplastic damage behavior of T700/PEEK. Finally, comparisons between the experimental tests and the numerical analysis were made, and a fairly good agreement was found, which validated the correctness of the proposed constitutive model in this work.

## 1. Introduction

Carbon fiber-reinforced polymer composites (CFRPs) have been integrated into many industrial applications for their structural parts with the requirement of a lightweight design and high reliability. Thermosetting resins, such as epoxy and others, have been widely used in various industry sectors. The thermoplastic resins, especially the polyetheretherketone (PEEK), have a higher impact resistance, better fracture toughness, and the advantage of waste recycling compared to the thermosetting resin [1,2,3]. Thus, the CFRPs of thermoplastic resins have great potential to be widely used in future aerospace, automotive, and civilian products. The stress–strain relationships of CFRPs with thermoplastic resins tend to be nonlinear and sensitive to the plastic strain [4,5], herein an accurate constitutive model is one of the most critical considerations in its structural integrity.

Numerous studies on constitutive behavior have been conducted on the thermosetting composites, whereas relatively few studies have been conducted on the thermoplastic composites. The research methods of the former can provide available guidance for the latter. It is widely accepted that the plastic potential plays a key role in the constitutive model due to its calculation of the effective stress of materials [6,7]. Based on a simple flow rule and the Hill plastic potential, Sun and Chen [8] first proposed a plastic potential by considering a state of plane stress. The plastic potential includes one undetermined parameter to characterize the level of plastic deformation developed under shear loading compared to transverse loading, and the single-parameter 2D plasticity model was employed to describe the nonlinear behavior of fibrous composites [9,10,11]. Sun and Chen [12] assumed afterward that the composites were transversely isotropic, and a 3D plastic potential was established with two undetermined parameters. It is always desirable to have fewer parameters in the constitutive model by comparing the two-parameter 3D plastic potential with the strain energy function for transversely isotropic elastic solids. Thus, two undetermined parameters in the 3D plastic potential were reduced to one by Weeks and Sun [13], and a single-parameter 3D model was established. Cho et al. [14] introduced a 3D plastic potential, combining the functions related to dilatational deformations, and a simplified 2D plastic potential with two unknown parameters was given to perform the numerical prediction. Also, the validating experiment of unidirectional carbon/epoxy (AS4/3501-6) was carried out in this study. Chen and Suo [15] constructed an elastoplastic constitutive model to describe the nonlinear compressive stress–strain behavior of the fiber-reinforced polymer composites (FRPs) of T800H/3631 and HTS40/PA6, and the model was developed under the single-parameter 2D plastic potential. T700/PPEK is a high-performance CFRP of thermoplastic resins. Yao et al. [16] carried out a numerical and experimental study to investigate the effect of interference fit on the bearing strength of the T700/PEEK riveted joint. The single-parameter 2D plastic potential was chosen, and the unknown single parameter was obtained using the off-axis test. No works were seen in the open literature on combining the single-parameter 3D model with the functions related to the dilatational deformations for the simulation of the elastoplastic behavior for the CFRPs, and no similar works were seen for the CFRPs of thermoplastic resins.

The hardening laws were utilized to express the master effective stress-plastic strain relation, and the unknown coefficients in the hardening laws can be obtained by performing the off-axis tensile tests [17,18]. Ding et al. [19] performed a 3D elastoplastic analysis of the interlaminar stresses for the AS4/PEEK composite laminate by using a single-parameter 3D plastic potential, and the master effective stress-plastic strain relation was fitted with a power-law function. An elastoplastic damage model considering the cohesive matrix interface layers was proposed for the composite laminates of AS4/PEEK by Mandal and Chakrabarti [20]. It was pointed out that a hardening was considered in the shear direction, and there were two shear hardening parameters given in this study. Chen and Suo [15] concluded that the effective plastic strain could be expressed by the effective stress, initial elastic response stress, and two hardening parameters, which assume that the hardening behavior obeys a power law with an index independent of fiber orientation, tension, and compression. However, further improvements can be made by changing the functional form to enhance the accuracy of the numerical prediction. Vyas et al. [21] developed an exponential strain-hardening model with an explicit integration finite element code and studied the nonlinear mechanical response of AS4/55A under different transverse loads, showing a good agreement between the simulation and experimental results. A pressure-dependent elastoplastic constitutive model was established for the unidirectional CFRP laminates of IM7/8552 and AS4/PEEK by Ren Rui et al. [22]. The hardening law was expressed by a piecewise function composed of four terms, including three exponential functions, the curves of predictions agreed well with the tests, but there were seven unknown coefficients to be determined in this law. These functional forms of hardening law in the aforementioned literature were generally obtained using the simple linear combinations of exponential functions. The relation of the dependent variable–independent variable in the Langmuir isothermal adsorption function [23,24,25] shows similar patterns to that of the effective stress-effective plastic strain in the constitutive model. In addition, there is a lack of research on the application of the Langmuir function in the hardening law for the CFRPs of both thermosetting and thermoplastic resins.

Finite Element Analysis (FEA) plays a crucial role in the development of composite materials, which have been widely embraced by the industry and academic research. [26,27]. Chen et al. [6] developed a user-defined subroutine within ABAQUS/Standard to analyze the plastic behavior of composite materials. This subroutine incorporates a single-parameter plastic potential, Hashin failure criteria, and exponential damage evolution to predict the plastic behavior, damage initiation, and failure process of AS4/PEEK. Liu et al. [18] examined the three-point bending behavior of AS4/PEEK through a comprehensive approach involving both experimental and numerical methods. In addition to conducting experimental research, a numerical model was constructed to enhance the understanding of damage mechanisms and evolution. Din et al. [28] developed a UMAT subroutine in ABAQUS/Standard finite element software to investigate the mechanical properties of open-hole laminates, considering elastic-plastic continuous damage. The study utilized a one-parameter flow rule for orthotropic plasticity to capture the nonlinear behavior of the fiber-reinforced composite with a thermoplastic matrix. The Puck theory was employed as the failure criterion, along with an exponential damage evolution rule to predict damage onset and evolution. The analysis results demonstrate a notable enhancement in predicting the mechanical behavior of continuous unidirectional composite materials. Harpreet et al. [29] proposed an elastoplastic damage model for 3D fiber-reinforced plastic (FRP) composites to simulate progressive damage and induced inelastic deformation resulting from low-velocity impacts. The model utilized 3D plastic potential for plastic surface growth and implemented an exponential softening model for predicting damage growth. Additionally, delamination behavior was considered by incorporating surface-based cohesive interactions between adjacent plies. The results demonstrated a close match between the predicted behavior and the experimental observations. In recent years, numerous failure criteria have been proposed, with the LaRC05 theory being recognized as one of the most accurate based on the second World-Wide Failure Exercise (WWFE-II). Wang et al. [30] developed a progressive damage model using the 3D LaRC failure criterion in conjunction with cohesive elements. They utilized an energy-based damage evolution method to forecast the behavior of single-lap thin-ply laminated composite-bolted joints under tension loading. Ma et al. [31] conducted a series of compression tests using specimens at different off-axis angles to study the damage initiation and progression mechanisms in unidirectional AS4/PEEK composites. The off-axis compression failure envelope, evaluated based on the LaRC05 criteria, was compared to the experimental results, demonstrating the ability of the LaRC05 criteria to predict failure accurately.

To overcome the aforementioned issues and limitations, the CFRPs of thermoplastic resins—T700/PEEK—were regarded as the research object. A two-parameter 3D plastic potential was given by combining the single-parameter 3D plastic potential with the function terms related to the dilatational deformations, induced from the hydrostatic pressure. The Langmuir function was first chosen to express the hardening law of CFRPs. Furthermore, off-axis tensile tests were conducted to obtain the unknown parameters both in the plastic potential and the hardening law. Finally, the prediction of numerical simulations, considering the 3D elastoplastic damage behavior, was compared to the results of the off-axis test to validate the availability of the proposed constitutive model.

## 2. Experiment Design and Results Analysis

### 2.1. Material and Specimen

**Figure 1:**
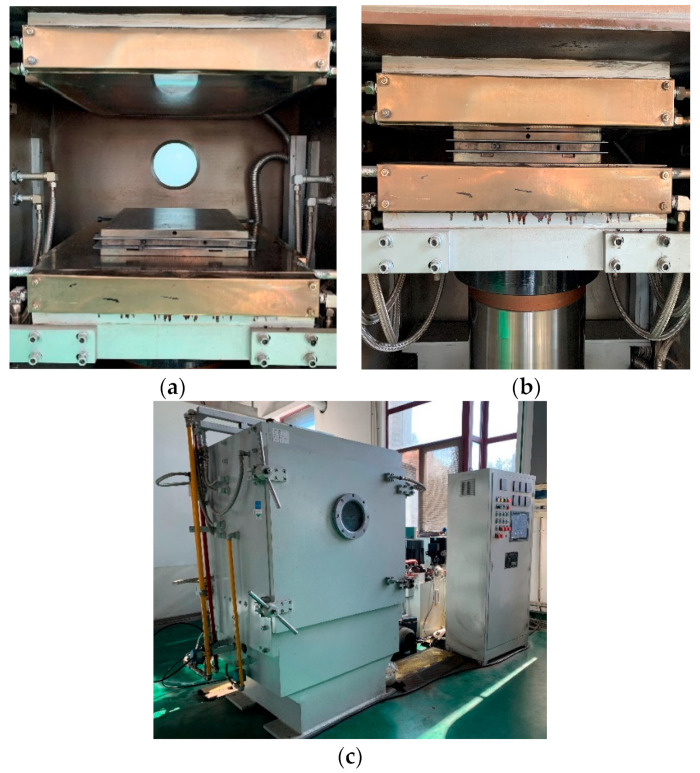
Main process of specimen preparation: (**a**) Mold opening. (**b**) Mold closing. (**c**) Vacuum sulphuration.

The unidirectional thermoplastic composites used in this study was T700/PEEK thermoplastic prepreg produced by Ningbo Material Technology and Engineering Research Institute, Ningbo, Zhejiang. It was fabricated by lying prepregs in a mental mold and then curing in a vacuum sulphuration at 380 °C and 3 MPa, as shown in Figure 1. There are seven samples with different fiber layups to be manufactured, namely [0]_8_, [15]_20_, [30]_20_, [45]_20_, [60]_20_, [75]_20_, and [90]_16_. After cooling to room temperature, the specimens were cut to have off-axis angles, with respect to the fiber direction. The test specimens were cut by using a five-axis linkage ultra-high pressure water jet cutting machine at 27 MPa and 800 mm/min to ensure that the samples did not have serious cutting damage or interlayer delamination. The size of test specimens for the [0]_8_ layup is 250 mm × 15 mm × 1 mm, the size of test specimens for the [90]_16_ layup is 175 mm × 25 mm × 2 mm, and the size of test specimens for the other layups is 250 mm × 25 mm × 2.5 mm. Reinforcement plates are attached to both ends of the test specimen, the size of reinforcement plates for the [0]_8_ layup is 56 mm × 15 mm × 2 mm, and the size of reinforcement plates for the other layups is 25 mm × 25 mm × 2 mm. At least five specimens were tested for each sample.

### 2.2. Off-Axis Testing and Results

**Figure 2:**
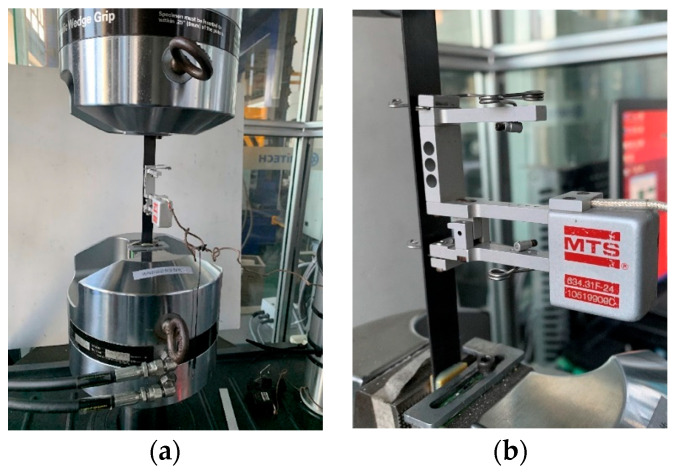
Experimental equipment for the off-axis tensile testing: (**a**) Overall diagram of the testing machine. (**b**) Specimen and extensometer.

The experiment was conducted using the universal testing machine (MTS 370 Load Frame; MTS Systems Corp., Eden Prairie, MN, USA), as shown in Figure 2, and its actuator force capacity was 100 kN. An extensometer with a 50 mm gauge length was equipped with the machine for specimen tests. Off-axis tensile tests were carried out according to the standard ASTM D3039 [32] and the loading was applied under displacement control at a rate of 2 mm/min. The frequency of data acquisition was set at 20 Hz.

The experimental data was processed according to ASTM D3039. The calculation formula for tensile stress is given by:

(1) $$\sigma_{i}=\frac{P_{i}}{A}$$

where *σ_i_* denotes the tensile stress of the *i*th data point, *P_i_* is the load of *i*th data point, and *A* represents the average cross-sectional area of the specimen.

The formula for calculating the elastic modulus is:

(2) $$E^{chord}=\Delta \sigma /\Delta \varepsilon$$

in which *E^chord^* refers to the tensile chord modulus of elasticity; Δ*σ* is the deviation value of applied tensile stress between two strain points on the extensometer; and Δ*ε* represents the deviation value of strain between two strain points.

**Figure 3:**
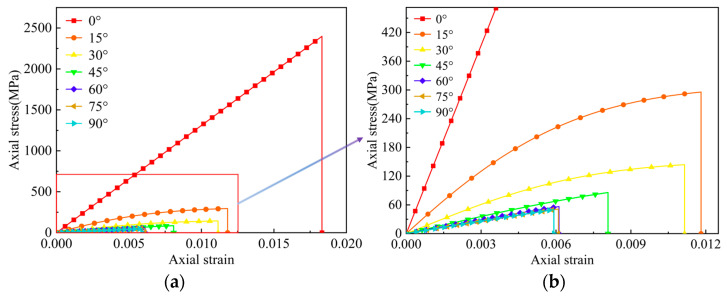
Axial tensile stress–strain curves: (**a**) The overall diagram. (**b**) The local diagram.

Let the direction of uniaxial tensile loading be the *x*-axis, which is parallel to the longitudinal direction of rectangular specimens, and the average experimental axial stress–strain (*σ_x_ − ε_x_*) curves of the off-axis tensile test are shown in Figure 3. From the stress–strain curves, we can see that when the off-axis angle is 0°, the mechanical behavior of the specimens is linearly elastic before its failure. As the off-axis angle gradually increases, the nonlinear behavior and ultimate fracture strain of specimens both show a decreasing trend. When the off-axis angle ranges from 60° to 90°, the mechanical behavior of the specimens only exhibits a small amount of approximately linearly elastic behavior, and then fracture failure occurs without apparent plastic deformation. This is because when the off-axis angle is large, the fiber cannot bear the majority of applied loads, resulting in the fracture failure of the matrix prior to the exhibition of plastic deformation.

**Figure 4:**
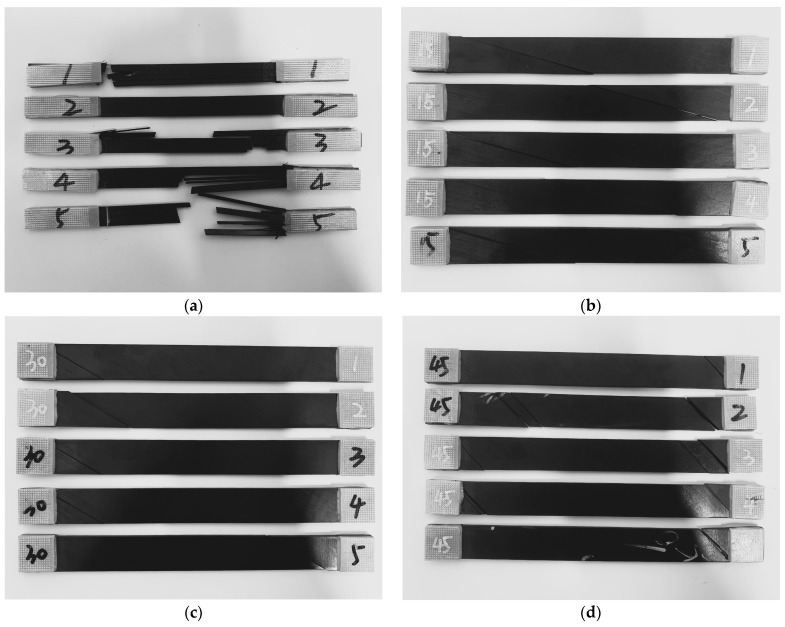

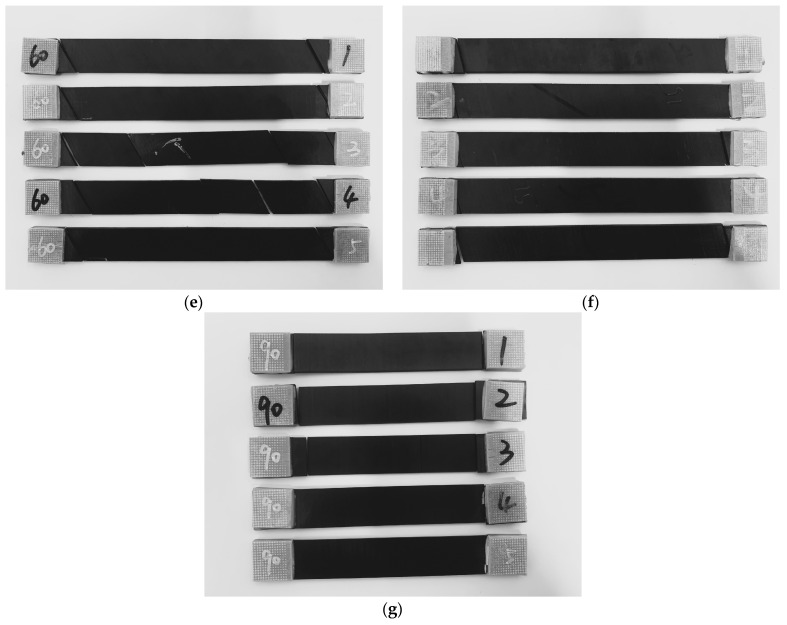
Specimens with off-axis tension fracture failure: (**a**) 0°. (**b**) 15°. (**c**) 30°. (**d**) 45°. (**e**) 60°. (**f**) 75°. (**g**) 90°.

The fracture surface angles are shown for the test specimens with different layups in Table 1. Due to the tensile failure mode of longitudinal splitting and fiber breakage in sample [0]_8_, the failure angle is set as zero. In terms of the off-axis angles among different specimens, the fracture surface angles increase accordingly, approximately the same value as the off-axis angle of the sample. The tested specimens with off-axis tension fracture failure are shown in Figure 4.

## 3. Constitutive Model Coupling with 3D Elastoplastic Damage

From the results of the off-axis tensile tests, it can be seen that the CFRPs of T700/PEEK laminates exhibit a significant nonlinear mechanical response and typical tensile failure mode due to plastic deformation under the off-axis tensile load. In order to simulate the above mechanical behavior accurately, a 3D elastoplastic constitutive model of the CFRPs of thermoplastic resins was established by considering the elastic deformation, plastic deformation, damage initiation, and damage evolution.

### 3.1. Elasticity Description

By assumption of the transverse isotropy for unidirectional composite laminates, the expression of stress **σ** during the linear elastic stage is:

(3) $$\sigma =C_{0}:\varepsilon^{e}$$

where ***C***_0_ represents the stiffness matrix of the initial undamaged composite laminates; **ε***^e^* denotes the elastic strain tensor, and Equation (3) can be specifically given by:

(4) $$\left[\begin{array}{c} \sigma_{11}\\ \sigma_{22}\\ \sigma_{33}\\ \sigma_{12}\\ \sigma_{23}\\ \sigma_{13} \end{array}\right]=\left[\begin{array}{cccccc} C_{11} & C_{12} & C_{13} & 0 & 0 & 0\\ C_{12} & C_{22} & C_{23} & 0 & 0 & 0\\ C_{13} & C_{23} & C_{33} & 0 & 0 & 0\\ 0 & 0 & 0 & C_{44} & 0 & 0\\ 0 & 0 & 0 & 0 & C_{55} & 0\\ 0 & 0 & 0 & 0 & 0 & C_{66} \end{array}\right]\left[\begin{array}{c} \varepsilon_{11}^{e}\\ \varepsilon_{22}^{e}\\ \varepsilon_{33}^{e}\\ \varepsilon_{12}^{e}\\ \varepsilon_{23}^{e}\\ \varepsilon_{13}^{e} \end{array}\right]$$

and

(5) $$\left\{\begin{array}{c} C_{11}=\lambda E_{11}(1-\nu_{23}\nu_{32})\\ C_{22}=\lambda E_{22}(1-\nu_{13}\nu_{31})\\ C_{33}=\lambda E_{33}(1-\nu_{12}\nu_{21})\\ C_{12}=\lambda E_{12}(\nu_{21}+\nu_{23}\nu_{31})\\ C_{23}=\lambda E_{22}(\nu_{32}+\nu_{12}\nu_{31})\\ C_{13}=\lambda E_{11}(\nu_{31}+\nu_{21}\nu_{32})\\ C_{44}=G_{12}\\ C_{55}=G_{23}\\ C_{66}=G_{13} \end{array}\right.$$

(6) $$\lambda =\frac{1}{1-\nu_{12}\nu_{21}-\nu_{32}\nu_{23}-\nu_{13}\nu_{31}-2\nu_{13}\nu_{32}\nu_{21}}$$

where the parameters *E_ij_* (*i*, *j* = 1, 2, 3, *i* = *j*) are the Young’s modulus; *ν_ij_* (*i*, *j* = 1, 2, 3, *i* ≠ *j*) are the Poisson ’s ratios; *G_ij_* (*i*, *j* = 1, 2, 3, *i* ≠ *j*) are the shear modulus. *σ_ij_* (*i*, *j* = 1, 2, 3) and $\varepsilon_{ij}^{e}$ (*i*, *j* = 1, 2, 3) are the stress and strain components, respectively.

### 3.2. Plasticity Description

For the test specimens, the applied external force is resisted by the effective and undamaged area of the material. Therefore, it can be reasonably assumed that plastic deformation occurs in the undamaged region of the material.

The plastic yield criterion proposed by Sun and Chen [8,12] can effectively simulate the plastic mechanical behavior of the composite laminates under quasi-static conditions. The form of this model is adopted:

(7) $$F(\sigma ,{\bar{\varepsilon }}^{p})=F^{p}(\sigma )-\bar{\sigma }({\bar{\varepsilon }}^{p})$$

where $F^{p}(\sigma )$ refers to the plastic potential; $\bar{\sigma }({\bar{\varepsilon }}^{p})$ represents the hardening function.

Weeks et al. [13] assumed that the composite is transversely isotropic and only linearly elastic deformation occurred in its fiber direction. Also, the effect of dilatational deformation on the plastic deformation was considered by Cho et al. [14]. By combining the single-parameter 3D plastic potential [13] and the dilatational function terms [14]. A two-parameter 3D plastic potential is then proposed:

(8) $$F^{p}(\sigma )=\sqrt{3[\frac{1}{2}(\sigma_{22}^{2}+\sigma_{33}^{2})-\sigma_{22}\sigma_{33}+2\sigma_{23}^{2}+a(\sigma_{12}^{2}+\sigma_{13}^{2})]}+b(\sigma_{22}+\sigma_{33})$$

where the values of *a* and *b* are both related to the properties of the composite material. As the dilatational deformations are ignored, namely *b* = 0, the proposed two-parameter 3D plastic potential in Equation (8) reduces to a single-parameter 3D plastic potential [13,33].

Defining effective stress as:

(9) $$\bar{\sigma }=F^{p}(\sigma )$$

Assuming that the plastic deformation of the materials satisfies the associated flow rule, the incremental component of plastic strain can be expressed by:

(10) $$d\varepsilon_{ij}^{p}=d\lambda \frac{\partial F^{p}(\sigma )}{\partial \sigma_{ij}},(i,j=1,\;2,\;3)$$

where *dλ* is the non-negative plastic multiplier of the entire plastic loading history; the gradient vector $\frac{\partial F^{p}(\sigma )}{\partial {\bar{\sigma }}_{ij}}$ represents the plastic gradient, which describes the direction of the plastic strain increment component $d\varepsilon_{ij}^{p}$.

Combining Equations (8) and (10), we obtain:

(11) $$\left[\begin{array}{l} d\varepsilon_{11}^{p}\\ d\varepsilon_{22}^{p}\\ d\varepsilon_{33}^{p}\\ d\varepsilon_{12}^{p}\\ d\varepsilon_{23}^{p}\\ d\varepsilon_{13}^{p} \end{array}\right]=d\lambda \frac{\partial F^{p}(\sigma )}{\partial {\bar{\sigma }}_{ij}}=d\lambda \left[\begin{array}{c} 0\\ 3(\sigma_{22}-\sigma_{33})/2\sqrt{\xi }+b\\ 3(\sigma_{33}-\sigma_{22})/2\sqrt{\xi }+b\\ 3a\sigma_{12}/\sqrt{\xi }\\ 6\sigma_{23}/\sqrt{\xi }\\ 3a\sigma_{13}/\sqrt{\xi } \end{array}\right]$$

(12) $$\xi =3[\frac{1}{2}(\sigma_{22}^{2}+\sigma_{33}^{2})-\sigma_{22}\sigma_{33}+2\sigma_{23}^{2}+a(\sigma_{12}^{2}+\sigma_{13}^{2})]$$

The hardening law is utilized to describe the yield stress evolution of the materials after entering the plastic phase. Sun and Chen [8,12] derived the effective plastic strain increment by defining the increment of effective plastic work per unit volume, *dW^p^*. They assumed that the product of effective stress and the increment of effective plastic strain is equal to the sum of the products of stress components in each direction and its corresponding increment of plastic strain. Finally, the relations can be given by:

(13) $$dW^{p}=\sigma_{ij}d\varepsilon_{ij}^{p}=\bar{\sigma }d{\bar{\varepsilon }}^{p}$$

Combining Equation (13) with Equations (9)–(12), it can be obtained that:

(14) $$\begin{array}{c} dW^{p}=\sigma_{ij}d\lambda \frac{\partial F^{p}(\sigma )}{\partial \sigma_{ij}}\\ =d\lambda \{\sigma_{11}\cdot 0+\sigma_{22}\cdot [\frac{3}{2\sqrt{\xi }}(\sigma_{22}-\sigma_{33})+b]+\sigma_{33}\cdot [\frac{3}{2\sqrt{\xi }}(\sigma_{33}-\sigma_{22})+b]\\ +\sigma_{12}\cdot \frac{3}{\sqrt{\xi }}a\sigma_{12}+\sigma_{23}\cdot \frac{6}{\sqrt{\xi }}\sigma_{23}+\sigma_{13}\cdot \frac{3}{\sqrt{\xi }}a\sigma_{13}\}=d\lambda [(\frac{1}{\sqrt{\xi }}\xi )+b(\sigma_{22}+\sigma_{33})]\\ =d\lambda \bar{\sigma }=\bar{\sigma }d{\bar{\varepsilon }}^{p} \end{array}$$

in which it is quite easy to find that:

(15) $$d\lambda =d{\bar{\varepsilon }}^{p}$$

Currently, many researchers [28,29] have concluded that the hardening laws for effective stress $\bar{\sigma }$ and effective plastic strain ${\bar{\varepsilon }}^{p}$ were obtained from experimental data. The following power hardening laws [34] are adopted:

(16) $$\bar{\sigma }({\bar{\varepsilon }}^{p})=A+B{\left({\bar{\varepsilon }}^{p}\right)}^{C}$$

In this study, the Langmuir function with three unknown parameters is introduced to characterize the hardening behavior of materials, and it can be defined as:

(17) $$\bar{\sigma }({\bar{\varepsilon }}^{p})=[\alpha \beta {\left({\bar{\varepsilon }}^{p}\right)}^{1-n}]/[1+\beta {\left({\bar{\varepsilon }}^{p}\right)}^{1-n}]$$

where *α*, *β*, and *n* are the material parameters that can be obtained through nonlinear fitting of the effective stress-plastic strain curves.

### 3.3. Damage Description

#### 3.3.1. Damage Initiation Criteria

In order to predict the initiation of various damage modes within a single layer and to evaluate the effective stress state during the loading process, this work uses the maximum stress criterion and the matrix failure criterion in the LaRC05 criterion to determine the damage initiation of fiber and matrix tension, respectively.

(1) Failure of the fiber tension (*F_ft_* ≥ 1, *σ*_11_ > 0):

(18) $$F_{ft}=\frac{\sigma_{11}}{X_{T}}$$

where *X_T_* represents the tensile strength in the fiber direction of unidirectional FRP laminates.

**Figure 5:**
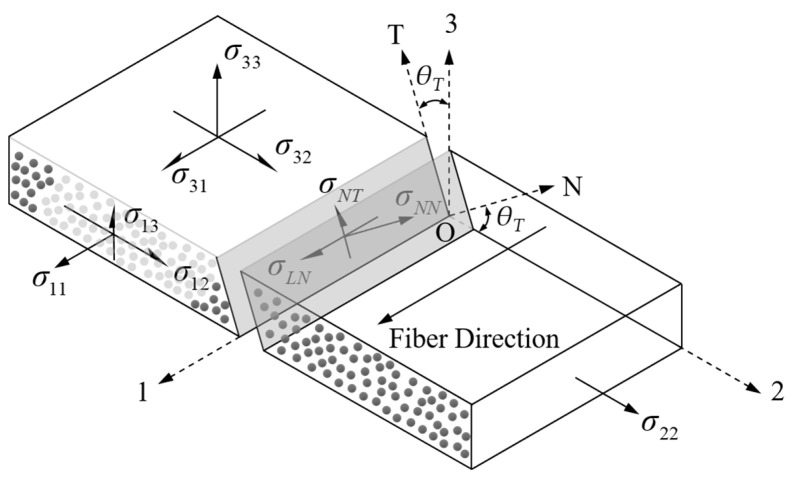
The stress state of a single layer in the composite materials and the stress components on potential fracture surfaces.

(2) Failure of the matrix tension (*F_mt_* ≥ 1, *σ_n_*(*θ_T_*) > 0):

Matrix failure, also known as inter-fiber failure, refers to the occurrence of initial fracture surfaces in the matrix of unidirectional FRP laminates under transverse stress and in-plane shear stress. The subsequent damage and failure behavior of the matrix will propagate along the fracture surfaces parallel to the fibers. As shown in Figure 5, the various stress components acting on the fracture surface of the matrix are given, where 1-2-3 represents the material coordinate system of the unidirectional lamina, L-N-T represents the local coordinate system of the fracture surface, and the 1-axis coincides with the L-axis. *θT* is the rotation angle between the normal direction of the potential fracture surface of the matrix and the 2-axis of the material coordinate system, and the range of rotation angle *θT* is [−90°, 90°]. *σNN*(*θT*) represents the normal stress on the fracture surface of the matrix, while *σNT*(*θT*) and *σLN*(*θT*) represent the shear stresses perpendicular and parallel to the fiber direction on the fracture surface of the matrix, respectively.

Pinho et al. [35,36] established a criterion to determine the initial damage of the matrix in unidirectional FRPs laminates, based on the Mohr–Coulomb fracture theory, using stress components in the local coordinate system of the initial fracture surface of the matrix.

(19) $$F_{mt}={\left(\frac{\sigma_{NT}(\theta_{T})}{S_{T}-\eta_{T}\sigma_{NN}(\theta_{T})}\right)}^{2}+{\left(\frac{\sigma_{LN}(\theta_{T})}{S_{L}-\eta_{L}\sigma_{NN}(\theta_{T})}\right)}^{2}+{\left(\frac{\sigma_{NN}(\theta_{T})}{Y_{T}}\right)}^{2}$$

In Equation (19), *S_T_* and *S_L_* represent the shear strengths perpendicular to the fiber direction and along the fiber direction, respectively, on the fracture surfaces of the matrix; *η_T_* and *η_L_* are the transverse shear and longitudinal shear friction coefficient of the matrix, respectively; *Y_T_* is the transverse tensile strength of the unidirectional FRPs laminate; and the relationship among *S_T_*, *S_L_*, *η_T_*, *η_L_*, and *Y_C_* can be given by:

(20) $$\left\{\begin{array}{c} S_{T}=\frac{Y_{C}}{2\tan\theta_{C}}\\ \eta_{T}=-\frac{1}{\tan2\theta_{C}}\\ \eta_{L}=\frac{S_{L}\eta_{T}}{S_{T}} \end{array}\right.$$

In Equation (20), *Y_C_* represents the transverse compressive strength of the unidirectional FRPs laminate; *θ_C_* denotes the angle between the fracture surface of the matrix and the 3-axis in the material coordinate system, as failure occurs under transverse compression. For CFRP composite laminates, researchers such as Puck et al. [37] have experimentally determined its range of values to be [51°, 55°].

The following relationship exists between the stress on the fracture surface and the initial fracture surface angle *θ_T_* of the matrix:

(21) $$\sigma_{\theta_{T}}=T_{\theta_{T}}\sigma$$

where:

(22) $$\sigma_{\theta_{T}}={\left[\sigma_{LL},\sigma_{NN},\sigma_{TT},\sigma_{LN},\sigma_{NT},\sigma_{LT}\right]}^{T}$$

(23) $$\sigma ={\left[\sigma_{11},\sigma_{22},\sigma_{33},\sigma_{12},\sigma_{23},\sigma_{13}\right]}^{T}$$

(24) $$T_{\theta_{T}}=\left(\begin{array}{cccccc} 1 & & & & & \\ & \cos^{2}\theta_{T} & \sin^{2}\theta_{T} & & 2\sin\theta_{T}\cos\theta_{T} & \\ & \sin^{2}\theta_{T} & \cos^{2}\theta_{T} & & -2\sin\theta_{T}\cos\theta_{T} & \\ & & & \cos\theta_{T} & & \sin\theta_{T}\\ & -\sin\theta_{T}\cos\theta_{T} & \sin\theta_{T}\cos\theta_{T} & & \cos^{2}\theta_{T}-\sin^{2}\theta_{T} & \\ & & & -\sin\theta_{T} & & \cos\theta_{T} \end{array}\right)$$

#### 3.3.2. Damage Evolution Law

When the internal stress of the material satisfies the failure criteria, the further increase in the effective stress will lead to the evolution of damage. During this process, strain energy is continuously released and the material exhibits localized softening phenomena, including the degradation of mechanical properties and a decrease in load-bearing capacity. By observing the experimental stress–strain curve of unidirectional T700/PEEK laminates, the curve segment concerning the damage evolution shows a sudden drop and degradation after reaching the ultimate tensile strength. In this study, a linear progressive degradation model is adopted to describe the damage evolution of the material [38,39].

(25) $$d_{I}^{\;t}=\max\{0,\min\{d_{I}^{\;*},\frac{\varepsilon_{I}^{final}(\varepsilon -\varepsilon_{I}^{0})}{\varepsilon (\varepsilon_{I}^{final}-\varepsilon_{I}^{0})}\}\};(I=ft,mt)$$

where $d_{I}^{\;t}$ represents the damage parameter at the current incremental step; *ε* is the equivalent strain in the composite ply and the strain values $\varepsilon_{I}^{0}$ and $\varepsilon_{I}^{\;final}$ are the equivalent strains corresponding to the initiation failure and final failure, respectively. When the material is undamaged, $d_{I}^{\;t}=0$; when the material is completely failed, $d_{I}^{\;t}=1$. In order to prevent the singularity of the material’s stiffness matrix due to stiffness reduction to zero during the finite element calculation process, $d_{I}^{\;*}=0.999$ is set.

The material strain is the direct cause of damage evolution, so when the strain energy release rate of the material is equal to its fracture toughness, the material has experienced complete failure. As the material model behaves softer, the damage of the material exhibits localizing characteristics as the dissipated energy decreases upon mesh refinement [40]. In order to avoid the effect of mesh size on the simulation results, Bazant’s crack band theory [41] is chosen in this work and l*, which represents the characteristic length of the element, is introduced for the numerical simulation analysis considering the linear progressive degradation. The energy released per unit volume *g_I_*_,*m*_ can be determined by the specific energy release rate *G_I_*_,*c*_ of the material.

(26) $$g_{I,m}=\frac{G_{I,c}}{l^{*}};(I=ft,mt)$$

The equivalent strains $\varepsilon_{I}$ of various damage modes at any given time, at the beginning of initial damage $\varepsilon_{I}^{0}$, and at complete failure $\varepsilon_{I}^{final}$, as well as the equivalent stresses $\sigma_{I}^{0}$ at the beginning of initial damage and the corresponding material fracture toughness calculation formula, are as follows:

(1) Fiber Tensile Failure:
  (27) $$\left\{\begin{array}{c} \sigma_{ft}^{0}=X_{T}\\ \varepsilon_{ft}=\varepsilon_{11}\\ \varepsilon_{ft}^{0}=\frac{X_{T}}{E_{11}}\\ \varepsilon_{ft}^{final}=\frac{2g_{ft,m}}{X_{T}} \end{array}\right.$$

For the fiber damage mode, the shear stress on the fracture surface is generally small. In this model, the consideration of equivalent strain is directly replaced by the strain in the direction of the fiber.

(2) Matrix tensile failure:

(28) $$\left\{\begin{array}{c} \left\{\begin{array}{c} \sigma_{mt}^{0}=\sqrt{{\langle \sigma_{NN}\rangle }^{2}+\sigma_{LN}^{2}+\sigma_{NT}^{2}}\\ \varepsilon_{mt}^{0}=\sqrt{{\langle \varepsilon_{NN}\rangle }^{2}+\varepsilon_{LN}^{2}+\varepsilon_{NT}^{2}} \end{array}\right.\\ \varepsilon_{mt}=\sqrt{{\langle \varepsilon_{NN}\rangle }^{2}+\varepsilon_{LN}^{2}+\varepsilon_{NT}^{2}}\\ G_{mt,c}=G_{IC}{\left(\frac{\langle \sigma_{NN}^{d}\rangle }{\sigma_{mt}^{0}}\right)}^{2}+G_{IIC}{\left(\frac{\sigma_{LN}^{d}}{\sigma_{mt}^{0}}\right)}^{2}+G_{IIIC}{\left(\frac{\sigma_{NT}^{d}}{\sigma_{mt}^{0}}\right)}^{2}\\ \varepsilon_{mt}^{final}=\frac{2G_{mt,c}}{\sigma_{mt}^{0}l^{*}} \end{array}\right.$$

where $\langle •\rangle$ represents the Macaulay operator, where $\langle x\rangle =(x+\left|x\right|)/2$ when *x* ∈ *R*. *G_IC_*, *G_IIC_*, and *G_IIIC_* refer to the fracture toughness corresponding to the opening-type I, sliding-type II, and tearing-type III cracks in the matrix, respectively. *ε_NN_* is the normal strain on the fracture surface of the matrix. *G_mt_*_,*c*_ is the equivalent fracture toughness of the matrix under mixed mode. *ε_NT_* and *ε_LN_* denote the shear strains that are perpendicular and parallel to the fiber direction on the fracture surface of the matrix, respectively. The calculation formulas are as follows:

(29) $$\varepsilon_{\theta_{T}}=T_{\theta_{T}}\varepsilon$$

where:

(30) $$\varepsilon_{\theta_{T}}={\left[\varepsilon_{LL},\varepsilon_{NN},\varepsilon_{TT},\varepsilon_{LN},\varepsilon_{NT},\varepsilon_{LT}\right]}^{T}$$

(31) $$\varepsilon ={\left[\varepsilon_{11},\varepsilon_{22},\varepsilon_{33},\varepsilon_{12},\varepsilon_{23},\varepsilon_{13}\right]}^{T}$$

Under the tensile loading, as *F_mt_* ≥ 1, the matrix damage occurs, which means that cracks occur in the matrix of the potential fracture surface. The mentioned damage can be simulated by reducing the traction component on the potential fracture surface, defined as follows:

(32) $$\left\{\begin{array}{c} \sigma_{NN}^{d}=(1-d_{mt})\sigma_{NN}\\ \sigma_{LN}^{d}=(1-d_{mt})\sigma_{LN}\\ \sigma_{NT}^{d}=(1-d_{mt})\sigma_{NT} \end{array}\right.$$

where $\sigma_{NN}^{d}$, $\sigma_{LN}^{d}$, and $\sigma_{NT}^{d}$ in Equation (32) represent the softened normal stress and shear stress on the potential fracture surface of the matrix, respectively. In this paper, we assume that the composite material is transversely isotropic and we defined *σ_TT_* and *σ_LT_* to be softened in the same manner as described by Equation (32). The softened stress in the global coordinate system can be given by:

(33) $$\sigma^{d}=T_{\theta_{T}}^{-1}\sigma_{\theta_{T}}^{d}$$

When the fiber reaches its ultimate tensile strength, its damage failure also affects the damage evolution of the matrix. Therefore, the stress reduction calculation formula caused by fiber tensile failure is defined as:

(34) $$\left[\sigma_{ij}^{d^{\prime }}\right]=\left[\begin{array}{c} (1-d_{ft})\sigma_{11}^{d}\\ \sigma_{22}^{d}\\ \sigma_{33}^{d}\\ (1-d_{ft})\sigma_{12}^{d}\\ \sigma_{23}^{d}\\ (1-d_{ft})\sigma_{13}^{d} \end{array}\right]$$

where $\sigma_{ij}^{d}$ and $\sigma_{ij}^{d^{\prime }}$ are the stresses after the softening of the material, corresponding to matrix fracture and fiber damage, respectively, in the global coordinate system.

## 4. Model Validation

### 4.1. Implementation of the Numerical Simulation

**Figure 6:**
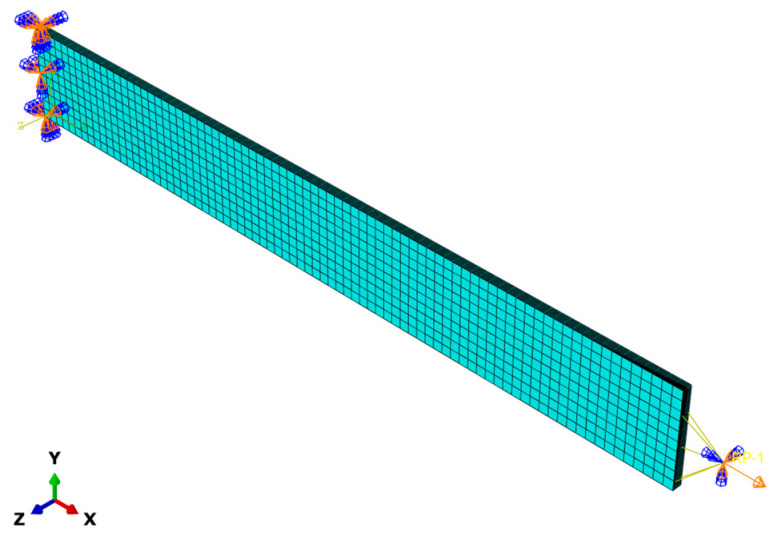
Finite element model of the off-axis tension tests for T700/PEEK laminated composites.

**Figure 7:**
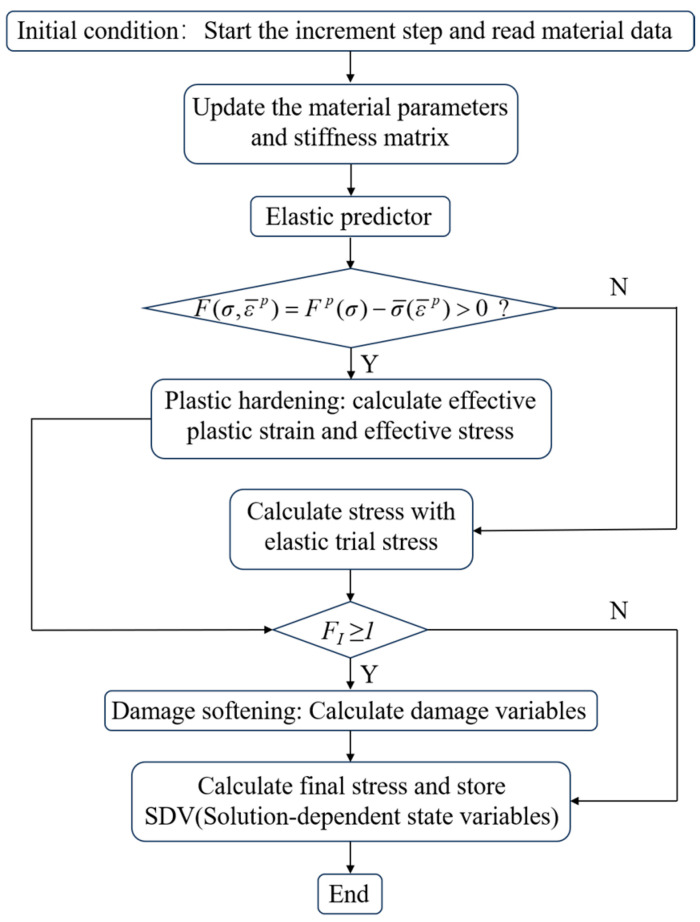
Flowchart of the User Subroutine (VUMAT).

As shown in Figure 6, the virtual specimen was discretized using eight-node linear reduced integration (C3D8R) solid elements with a size of 2.5 mm × 2.5 mm × 0.125 mm and assumes that the interfaces between layers are completely bonded. The left end of the finite element model is constrained to restrict the displacement in the loading direction and the right end loading is controlled by a displacement applied to a reference point outside its face. Computational accuracy was set as double precision to reduce the accumulation error during simulation. The proposed 3D elastoplastic damage constitutive model was implemented in ABAQUS 2021, using a user-defined material subroutine (VUMAT). A detailed flow chart of the VUMAT is presented in Figure 7. To validate the model, off-axis tensile analysis tests were simulated by using ABAQUS 2021. As shown in Table 2, the material parameters for T700/PEEK unidirectional laminates are presented, among which the fiber tensile fracture energy, matrix fracture energy, in-plane shear strength, and transverse compressive strength are taken from the literature [30,42,43]. The remaining material parameters are measured from the manufacturer’s experiments and are provided by the manufacturer.

### 4.2. Determination of the Plasticity Parameters

**Figure 8:**
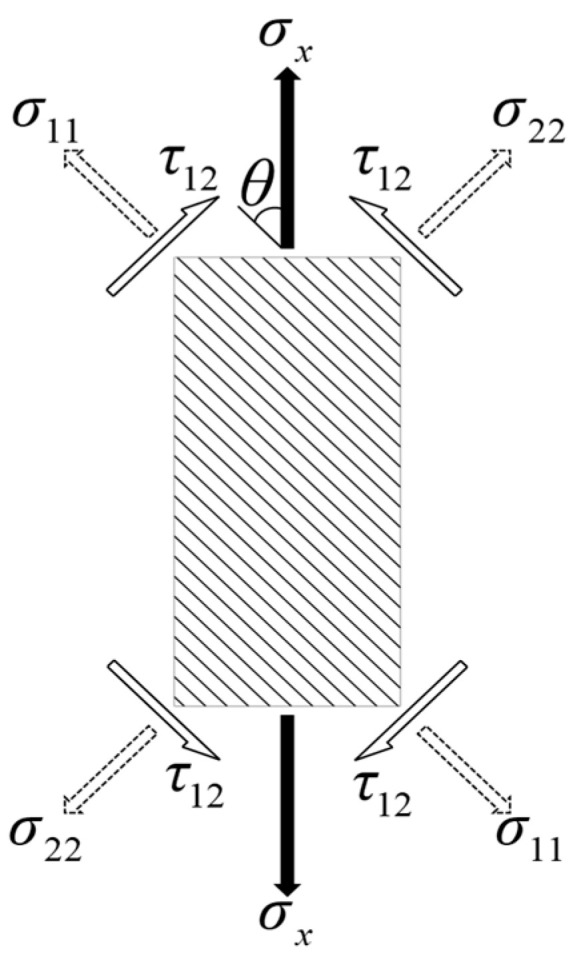
Unidirectionally reinforced composite laminates under longitudinal tensile loading.

The relationship between the effective stress and effective plastic strain for a given material is uniquely determined and is independent of loading conditions [44]. Therefore, the plastic parameters of the constitutive model in the plastic phase can be attained through longitudinal tensile tests. As shown in Figure 8, the composite laminate is subjected to a longitudinal tensile load, then a state of plane stress can be assumed.

The stress components are transformed along the principal material axes and are expressed in terms of axial stress (*σ_x_*), as follows:

(35) $$\sigma_{11}=\cos^{2}\theta \sigma_{x}$$

(36) $$\sigma_{22}=\sin^{2}\theta \sigma_{x}$$

(37) $$\sigma_{12}=-\sin\theta \cos\theta \sigma_{x}$$

where *θ* represents the angle between the tensile loading direction and the fiber direction, and the effective stress $\bar{\sigma }$ can be obtained by combining Equations (35)–(37) with Equation (8):

(38) $$\bar{\sigma }=h(\theta )\sigma_{x}$$

and the transformation function *h*(*θ*) can be given by:

(39) $$h(\theta )=\sqrt{\frac{3}{2}(\sin^{4}\theta +2a\sin^{2}\theta \cos^{2}\theta )}+b\sin^{2}\theta$$

Assuming that the studied material exhibits significant plastic deformation and small deformation. In terms of the experimentally measured stress–strain data, the incremental plastic strain $d\varepsilon_{x}^{p}$ can be obtained by subtracting the incremental elastic strain $d\varepsilon_{x}^{e}$ from the incremental strain *dε_x_*, and it can be expressed as:

(40) $$d\varepsilon_{x}^{p}=d\varepsilon_{x}-d\varepsilon_{x}^{e}$$

The incremental elastic strain $d\varepsilon_{x}^{e}$ can be computed from:

(41) $$d\varepsilon_{x}^{e}=\frac{d\sigma_{x}}{E_{x}}$$

The elastic modulus *E_x_* at different off-axis angles can be experimentally achieved by the stress–strain curve, and it also can be theoretically determined by the elastic modulus *E*_1_ and *E*_2_, off-axis angel *θ*, as well as the shear modulus *G*_12_ and Poisson ’s ratio *ν*_12_. The theoretical calculation formula can be given by:

(42) $$E_{x}=\frac{1}{\frac{1}{E_{1}}\cos^{4}\theta +\left(\frac{1}{G_{12}}-\frac{2\nu_{12}}{E_{1}}\right)\sin^{2}\theta \cos^{2}\theta +\frac{1}{E_{2}}\sin^{4}\theta }$$

**Figure 9:**
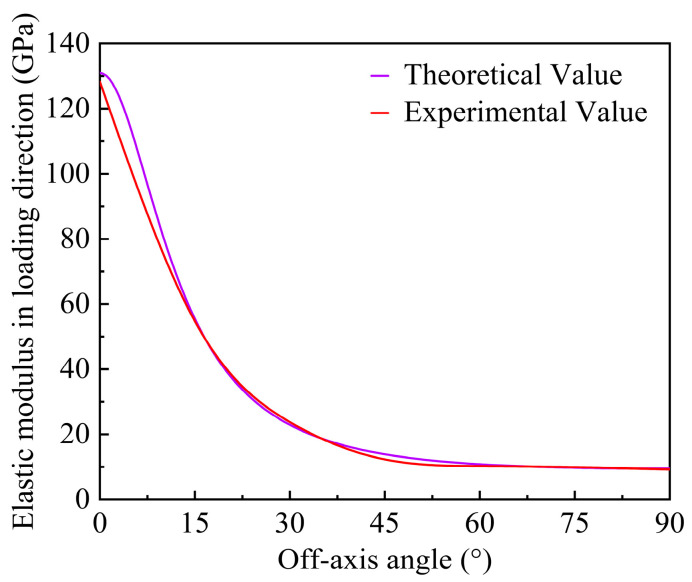
Comparison of the elastic modulus in loading direction between the theoretical and experimental values under different off-axis angles.

As shown in Figure 9, a comparison concerning the elastic modulus in loading direction is made between the experimental data and calculation value obtained by Equation (42). It can be observed that there is a comparatively good agreement between the experimental and theoretical values, which confirm the validity of the experiment in this work.

According to the coordinate transformation law of plastic strain components, the increment of plastic strain in the uniaxial loading direction can be expressed as [45]:

(43) $$d\varepsilon_{x}^{p}=\cos^{2}\theta d\varepsilon_{11}^{p}+\sin^{2}\theta d\varepsilon_{22}^{p}-\sin\theta \cos\theta d\varepsilon_{12}^{p}$$

Based on Equations (8), (10), (15) and (35)–(37), the effective plastic strain can be simplified as:

(44) $${\bar{\varepsilon }}_{p}=\frac{\varepsilon_{x}^{p}}{h(\theta )}$$

For the determination of plasticity parameters *a* and *b* in Equation (8), many researchers adopt artificial methods, like trial and error, to obtain them. In this study, an objective function regarding the plasticity parameters is provided to make each curve of effective stress-plastic strain associated with different off-axis angles coincide with one master curve. The plastic parameters *a* and *b* can be obtained by minimizing the objective function in Equation (45) by adopting the quasi-Newton algorithm. This aims to further enhance the consistency of the effective stress-plastic strain curves at different off-axis angles. The expressions are given as follows:

(45) $$f(a,b)=\sqrt{\sum_{{\bar{\varepsilon }}^{p}}{\left[{\bar{\sigma }}_{\theta_{1}}({\bar{\varepsilon }}^{p})-{\bar{\sigma }}_{\theta_{2}}({\bar{\varepsilon }}^{p})\right]}^{2}};\;(\theta_{1},\;\theta_{2}=15^{\circ },\;30^{\circ },\;45^{\circ },\;60^{\circ },\;75^{\circ },\;90^{\circ },\;\theta_{1}\neq \theta_{2})$$

where ${\bar{\sigma }}_{\theta }({\bar{\varepsilon }}^{p})$ is the effective plastic strain at different off-axis angles.

The plastic parameters were then calculated by using the quasi-Newton algorithm programmed in MATLAB R2021b. The initial and final values of the effective plastic strain were set to 10^−6^ and 0.01, respectively, with a step size of 10^−6^. The initial values of the plastic parameters were set to 0.5, and the optimization results of the objective function are provided in Table 3. It can be observed that the optimal objective function value for the two-parameter function model is 18,491.6292, whereas the optimal objective function value for the single-parameter function model is 31,926.5522. This indicates that the proposed approach is more suitable for characterizing the plastic behavior of CFRPs with thermoplastic resins.

**Figure 10:**
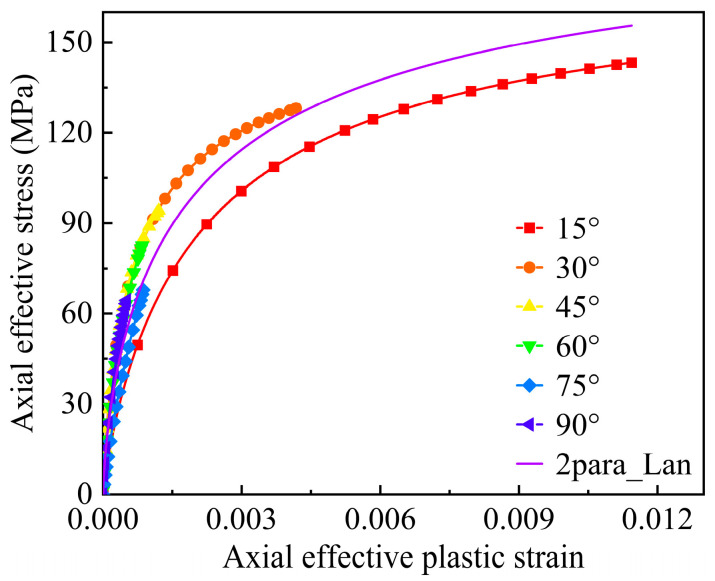
Fitting of the effective stress-plastic strain curve under off-axis tensile loading.

The power function of plastic hardening was regarded as the existing and generally adopted approach to characterizing the hardening law. In this work, the effective stress-plastic strain relationships are fitted by using the Langmuir function. According to Equations (38) and (44), the axial stress-plastic strain curves at different off-axis angles were transformed into effective stress-plastic strain curves, which were analyzed and fitted with a main curve by two-parameter Langmuir function (2 para_Lan) model, as shown in Figure 10. The processed data points show good consistency, and the parameters of the fitted hardening function are as follows: *α* = 200.01, *β* = 87.61, and *n* = 0.28.

### 4.3. Prediction of the Results and Verification

**Figure 11:**
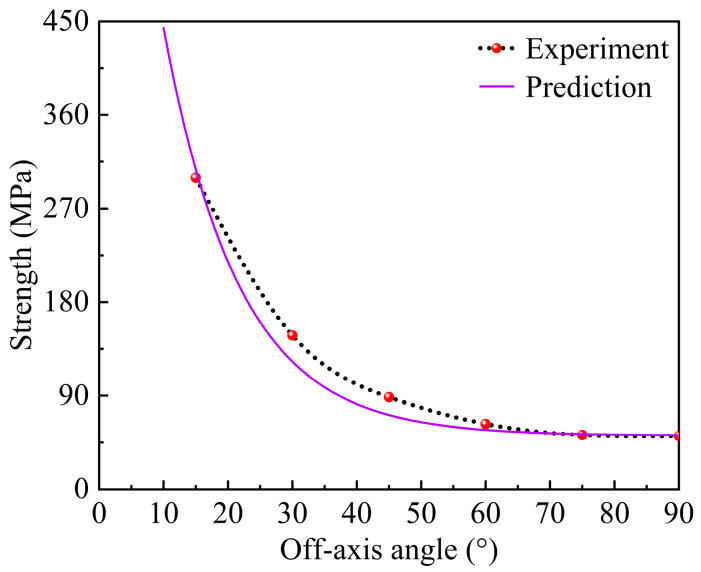
Comparison chart of the experimental and predicted tensile strength values at different off-axis angles.

**Figure 12:**
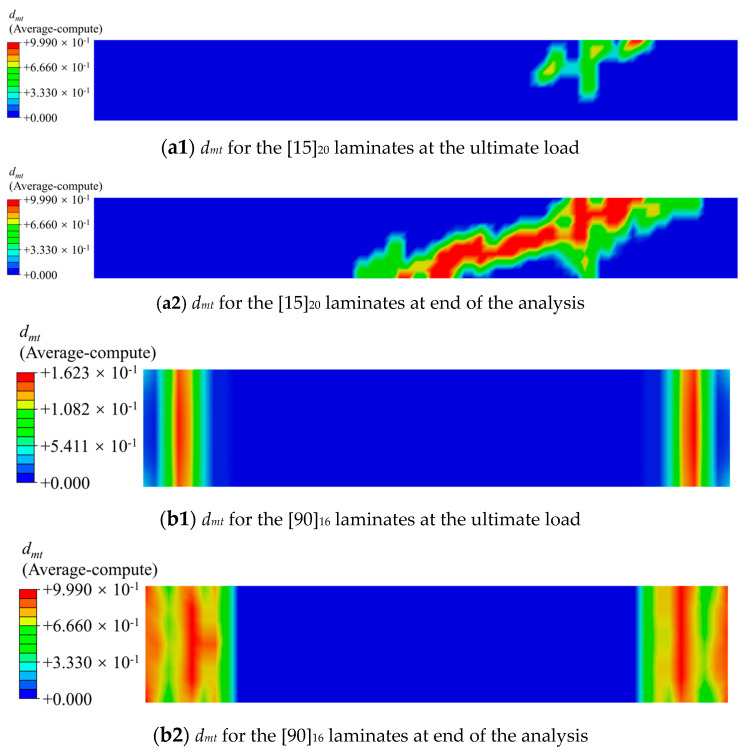
Plot of the matrix damage variables for [15]_20_ and [90]_16_ laminates.

As shown in Figure 11, the failure envelope curve, predicted by the present failure criterion, is in good agreement with the experiment data. Under the condition of the same loaded area of the specimen, the tensile strength decreases rapidly within the range of 0° to 15° of off-axis angle. However, as the off-axis angle further increases, the decreasing trend of the tensile strength gradually becomes gentler. The cloud map in Figure 12 shows the amount of matrix damage variables at the ultimate load and end of the analysis of the specimen, which was quantified by the variable of matrix damage *d_mt_*. As the *d_mt_* is 0, it indicates that the matrix has not been damaged, while when the *d_mt_* is 0.999, it indicates that the matrix has completely failed and has lost its bearing capacity. As is depicted in this cloud map, the matrix first suffers damage in the gage section of the specimen. With the further increase in the displacement load, the damage of the matrix begins to evolve towards the undamaged areas, and the evolution direction is consistent with the fiber direction. At the end of the analysis, the matrix damage has completely penetrated the cross-section of the specimen, directly leading to the complete failure and loss of load-bearing capacity of the specimen.

**Figure 13:**
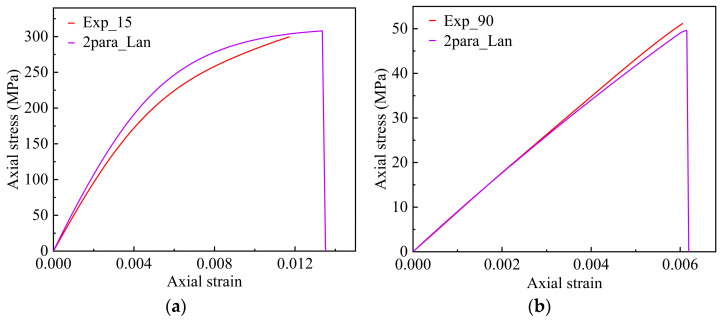
Comparison of the axial stress–strain curves between the experimental test and different prediction approaches under two off-axis angles: (**a**) 15°; (**b**) 90°.

**Figure 14:**
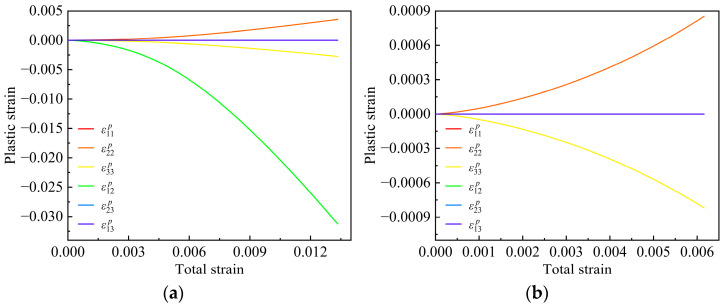
Tensile plastic strain-total strain curves at the different off-axis angles: (**a**) 15°; (**b**) 90°.

The stress–strain curves obtained from numerical simulations and experiments for the 15° and 90° layups are compared in Figure 13. It can be observed that the proposed model in this study can effectively predict the mechanical behavior of the unidirectional T700/PEEK composite materials. Figure 14 shows the plastic strain components-total axial strain relations under different off-axis angles. Due to the assumption that there is no plastic deformation in the fiber direction, $\varepsilon_{11}^{p}$ is always zero. The plastic strain components in the 1–3 and 2–3 plane, $\varepsilon_{13}^{p}$ and $\varepsilon_{23}^{p}$, are the minimum values among all the plastic strain components which can be almost negligible. The curves of the plastic strain components, $\varepsilon_{13}^{p}$ and $\varepsilon_{23}^{p}$, versus the total axial strain *ε* both like a straight line almost perpendicular to the vertical axis, and the two lines almost coincide with each other. It can be observed that the plastic strain components $\varepsilon_{12}^{p}$ plays a major contribution on the plastic deformation for the off-axis angles 15°. In particular, it can still be observed that the in-plane shear plastic strains generated are approximately 9 times and 12 times larger in the 2 and 3 directions, respectively. This indicates that plastic deformation is predominantly governed by the in-plane shear strain. However, as the off-axis angle increases further, accompanied by the weakening of in-plane plastic shear effects, the material’s nonlinear behavior also gradually weakens. When the off-axis angle increases to 90°, the in-plane plastic shear strain decreases to zero, and the plastic behavior of the material is mainly contributed to by the plastic strains in the 2 and 3 directions.

## 5. Conclusions

(1) By conducting off-axis tensile tests on seven types of samples, namely [0]_8_, [15]_20_, [30]_20_, [45]_20_, [60]_20_, [75]_20_, and [90]_16_, the corresponding axial stress–strain curves, elastic modulus, and tensile strengths were obtained. The fracture surface angles of the specimens for different off-axis angles were measured by using a high-precision digital angle gauge, and the difference between the measured fracture surface angle and the off-axis angle of each specimen was within −2°~3°. This difference is small and acceptable, and it may be caused by the manufacturing defects of the unidirectional T700/PEEK specimen.
(2) A two-parameter 3D plastic potential was developed by incorporating both deviatoric and dilatation deformations. The plastic parameters in the proposed plastic potential are determined using the quasi-Newton algorithm to optimize an objective function related to these plastic parameters. The Langmuir function was initially employed to fit the effective stress–strain relationships of the composites for hardening behavior, providing a precise prediction of the nonlinear mechanical response in CFRPs with thermoplastic resins.
(3) The two-parameter 3D plastic potential was integrated with the Langmuir function of plastic hardening to model the constitutive behavior for unidirectional T700/PEEK Laminates. Based on the proposed constitutive model, a user-defined subroutine (VUMAT), based on ABAQUS, has been developed. This subroutine incorporates the maximum stress criterion for fiber failure and the LaRC05 criterion for matrix failure, allowing for the simulation of 3D elastoplastic damage behavior during tensile loading tests. This work constructs a two-parameter 3D elastoplastic damage constitutive model considering the material plasticity, damage evolution, and dilatational deformation.
(4) Applying the 3D elastoplastic damage constitutive model to simulate the off-axis tensile behavior of unidirectional T700/PEEK laminates, the predicted stress–strain curves align closely with experimental data. This suggests that the model effectively captures the plastic behavior of unidirectional T700/PEEK thermoplastic laminates. The analysis of plastic strain in various directions indicates that shear plastic strain in the 1–2 plane governs plastic deformation. Nevertheless, this effect diminishes as the off-axis angle increases. The comparison between the numerically simulated and experimentally measured tensile strengths under identical loading planes reveals a gradual decrease in ultimate tensile strength with an increasing off-axis angle.

This work has investigated the nonlinear behavior of unidirectional thermoplastic composites under off-axis tensile loading. An anisotropic constitutive model for the nonlinear kinematic hardening elastoplastic damage of woven thermoplastic composites is still needed, including the asymmetry of tension and compression under cyclic loading conditions.

## Figures and Tables

**Table 1:** Fracture surface angles for the different layup methods.

Sample Number	Layup						
	[0]_8_	[15]_20_	[30]_20_	[45]_20_	[60]_20_	[75]_20_	[90]_16_
	Fracture Surface Angles/°						
Sample 1	0	15.06	29.35	47.45	60.05	75.90	90.05
Sample 2	0	14.95	30.03	43.45	61.35	75.45	92.00
Sample 3	0	15.70	29.45	46.10	60.32	75.54	89.70
Sample 4	0	14.75	29.65	44.90	60.45	74.00	89.55
Sample 5	0	15.35	30.30	45.60	60.24	76.35	89.25
Average value	0	15.16	29.76	45.50	60.48	75.45	90.11
Standard deviation	0	0.33	0.36	1.32	0.45	0.79	0.98

**Table 2:** The material parameters of the T700/PEEK.

*E*_11_ (GPa) *	*E*_22_ (GPa) *	*E*_33_ (GPa) *	*G*_12_ (GPa) *	*G*_13_ (GPa) *	*v*_12_ *
130	10.1	10.1	5.77	5.77	0.32
*v*_13_ *	*X_T_* (MPa) *	*X_C_* (MPa) *	*Y_T_* (MPa) *	*Y_C_* (MPa) [43]	*S_L_* (MPa) [43]
0.32	2419	1094	50.2	205.9	133
*G_ft_* (KJ/m^2^) [30]	*G_I_* (KJ/m^2^) [42]	*G_II_* (KJ/m^2^) [42]	*G_III_* (KJ/m^2^) [42]		
133	1.7	2.0	2.0		

* Values provided by the manufacturer.

**Table 3:** The calculation results of the optimization objective function between the single-parameter and two-parameter plastic potential.

Parameter	Plastic Potential	
	Single-Parameter	Two-Parameter
a	3.0727	1.2612
b	-	0.0252
Objective function value	31,926.5522	18,491.6292
Computational time/s	0.5690	0.9793

## Data Availability

The original contributions presented in the study are included in the article, further inquiries can be directed to the corresponding author.
